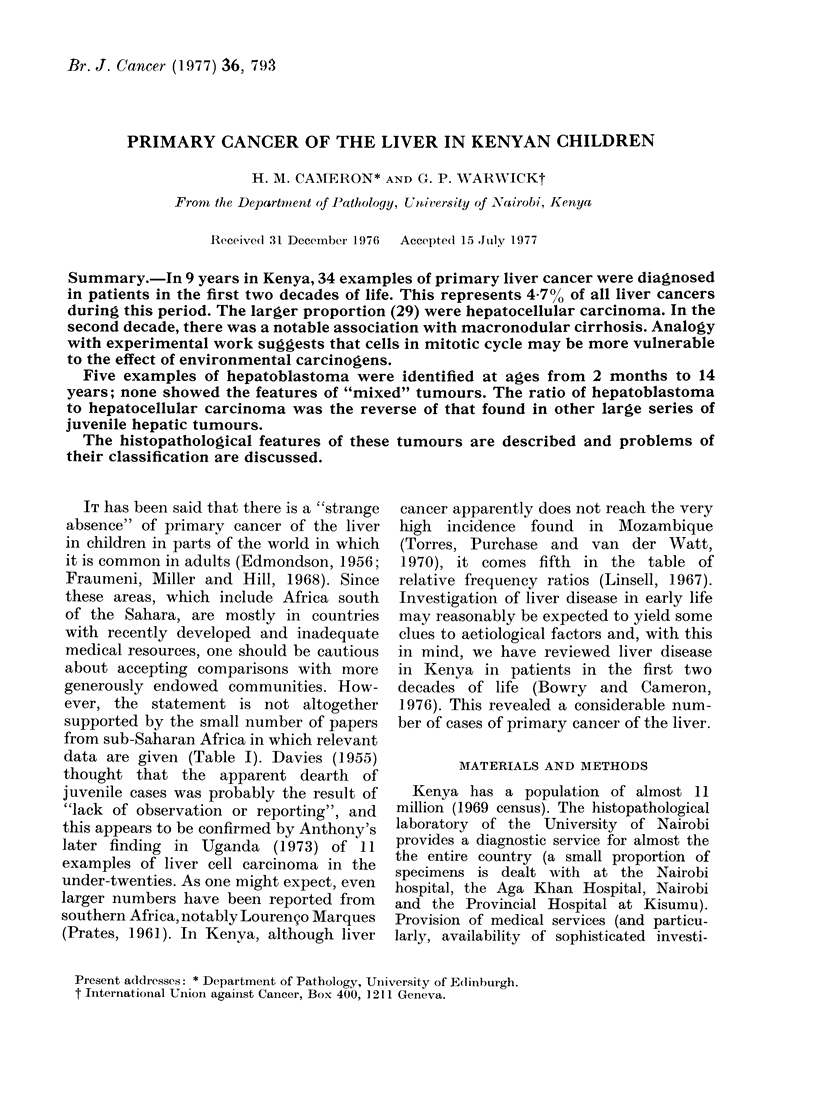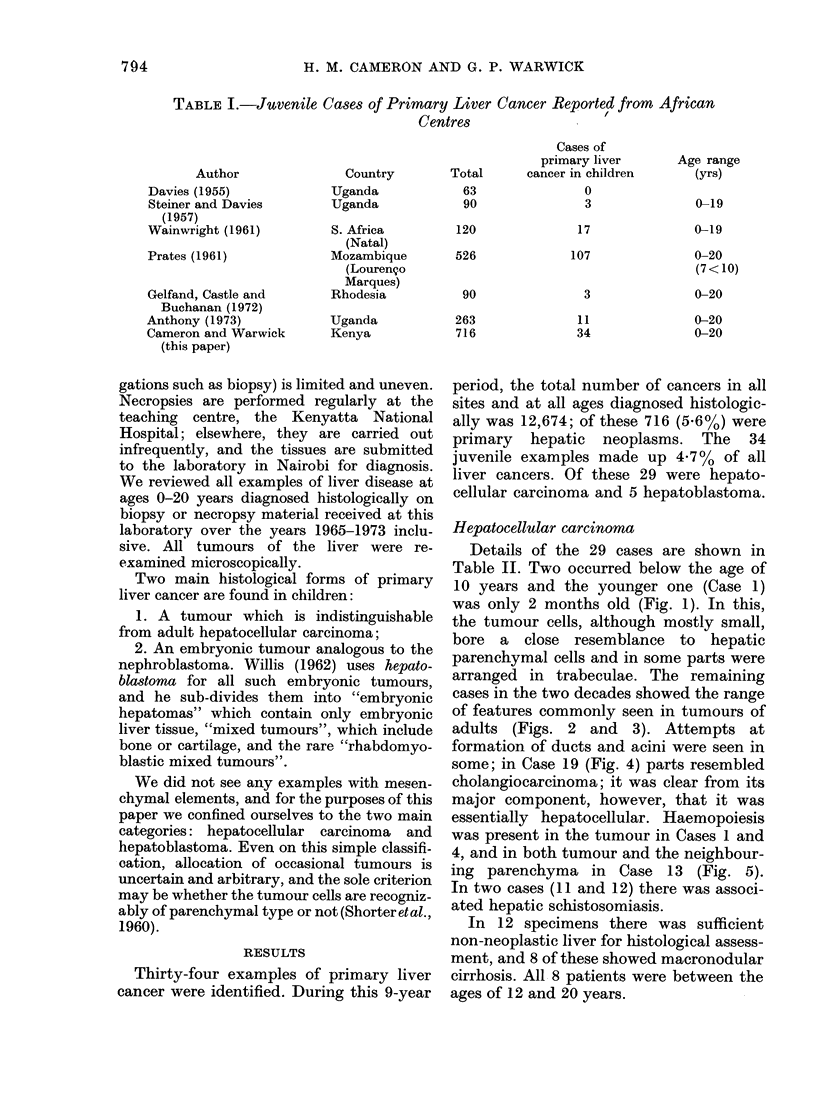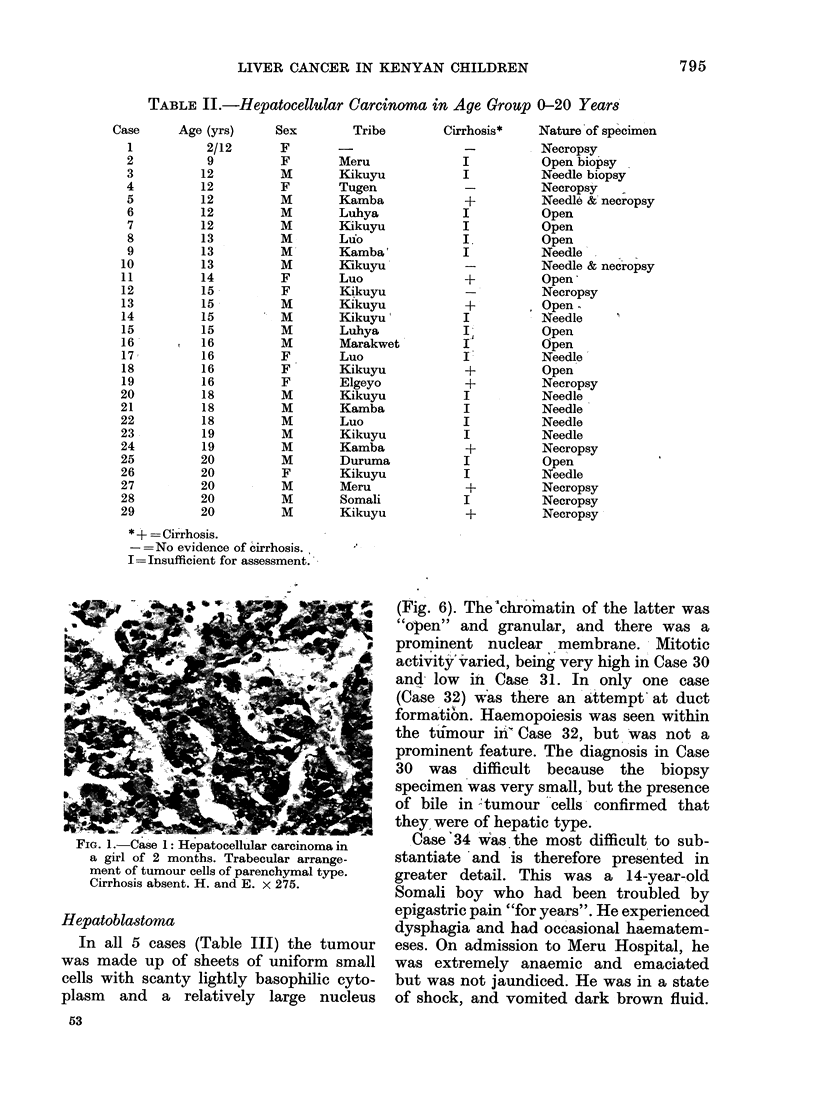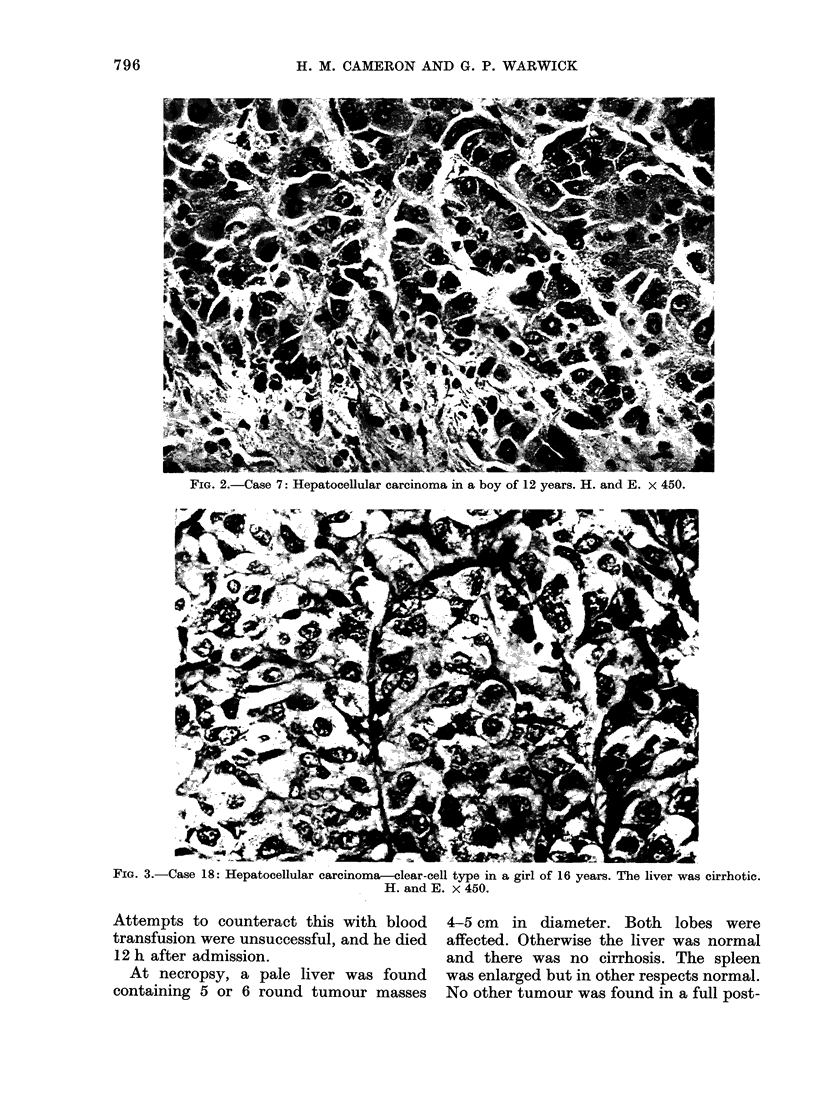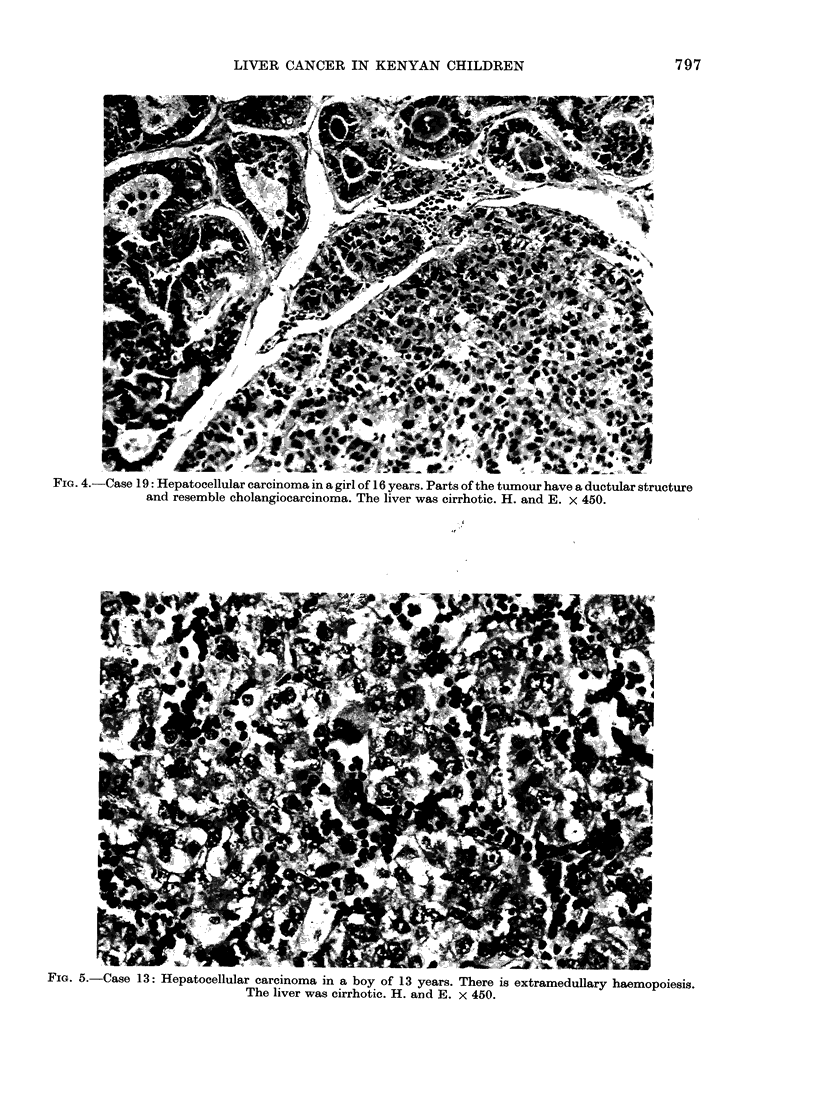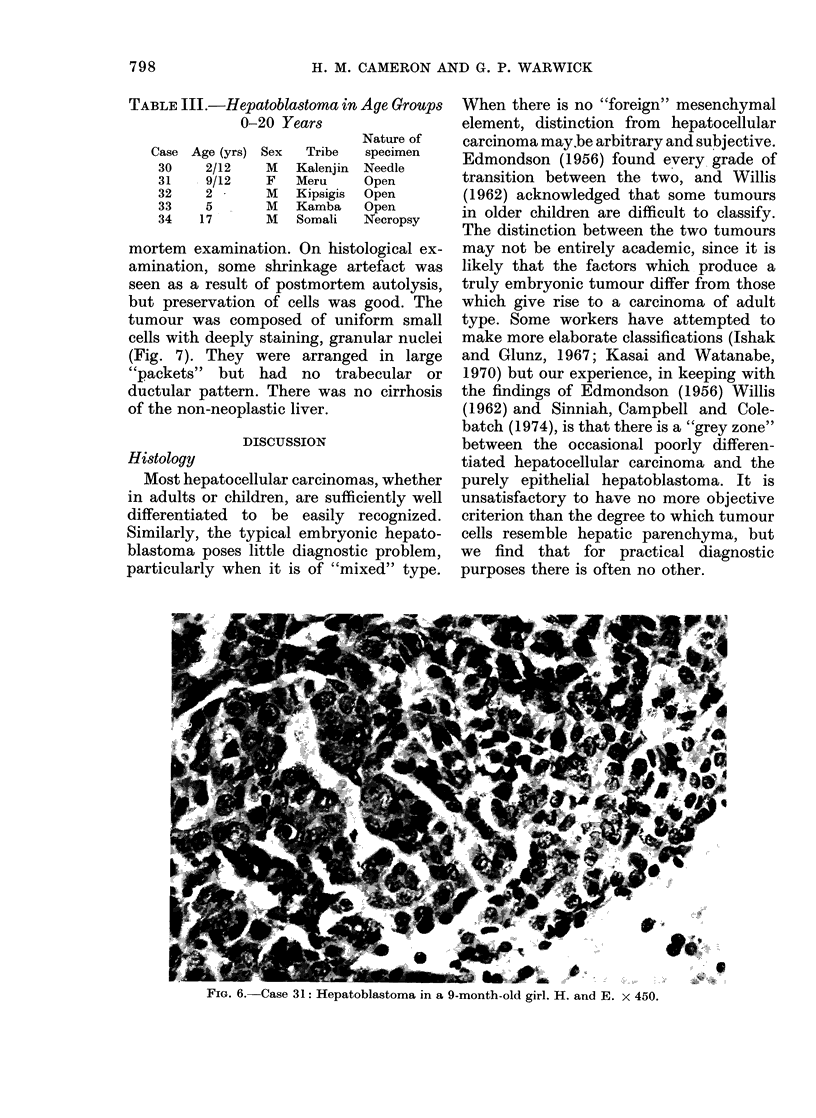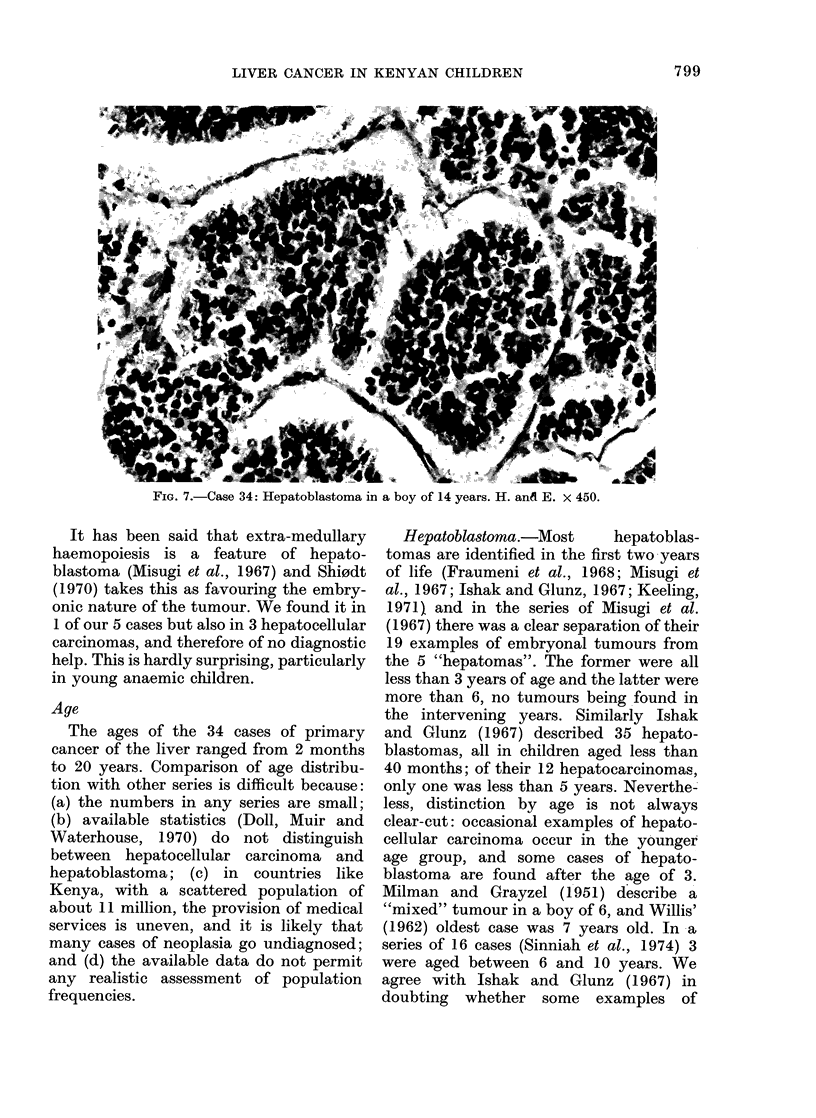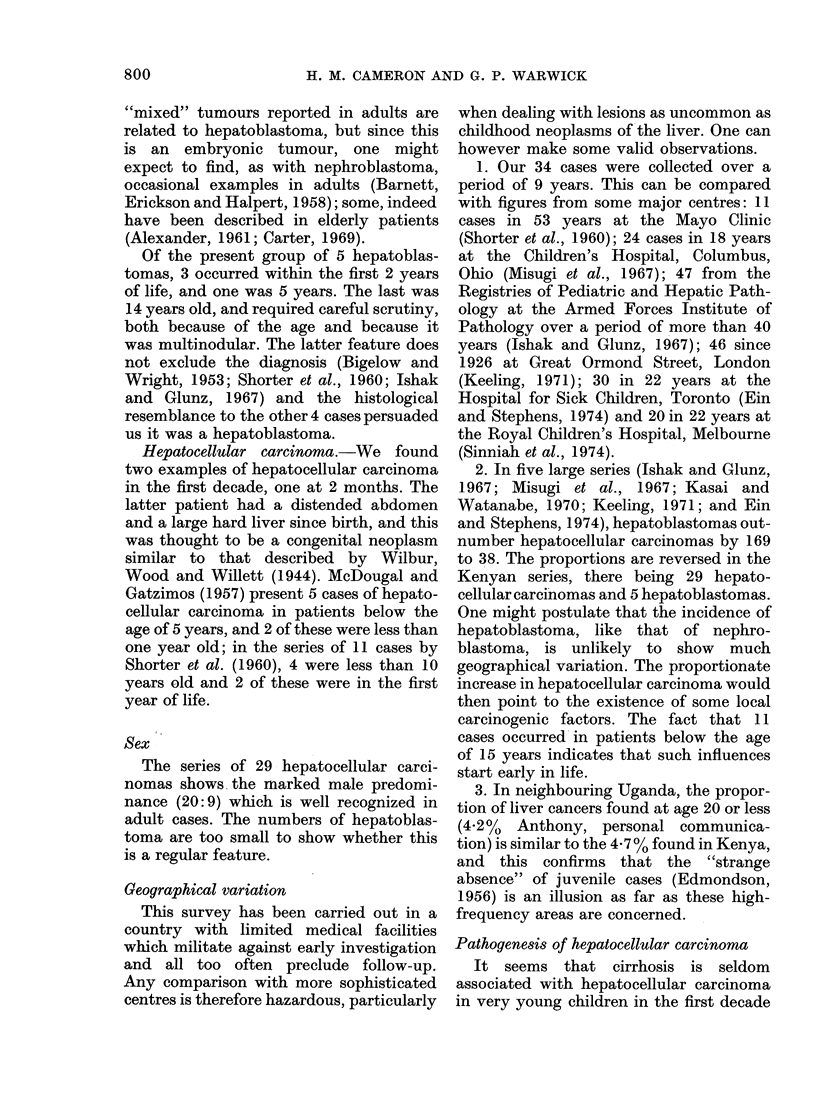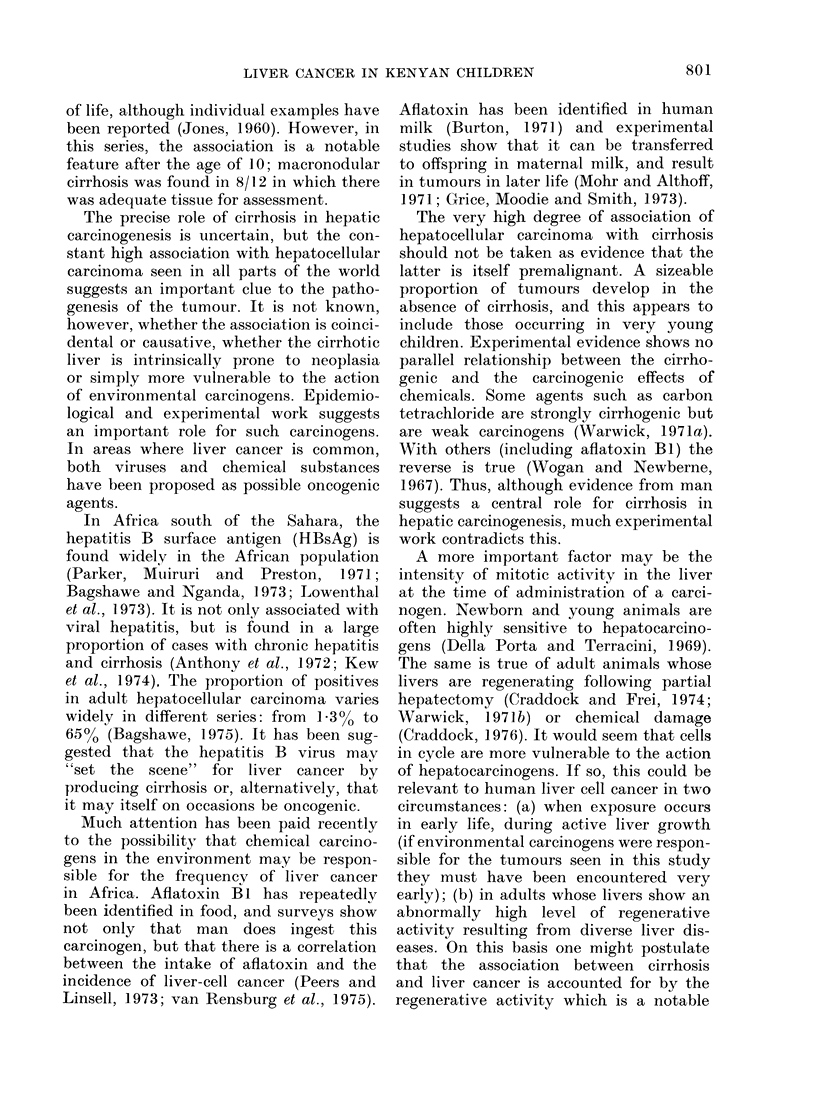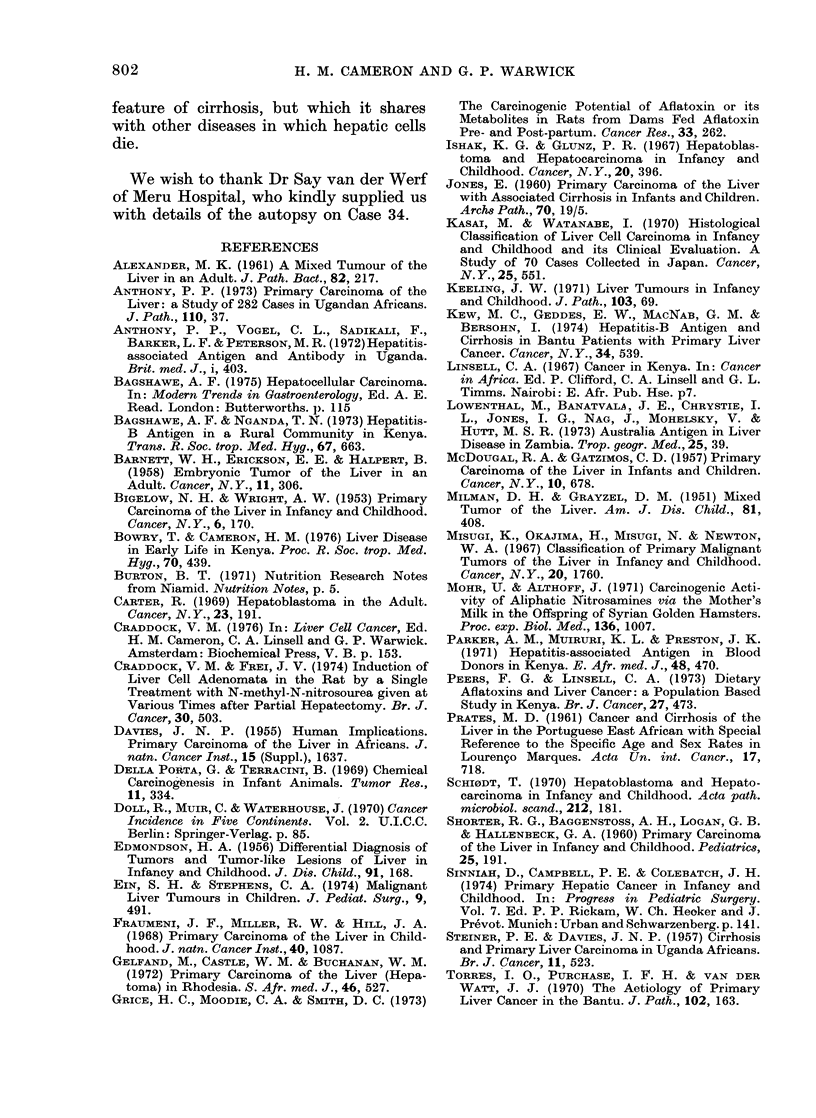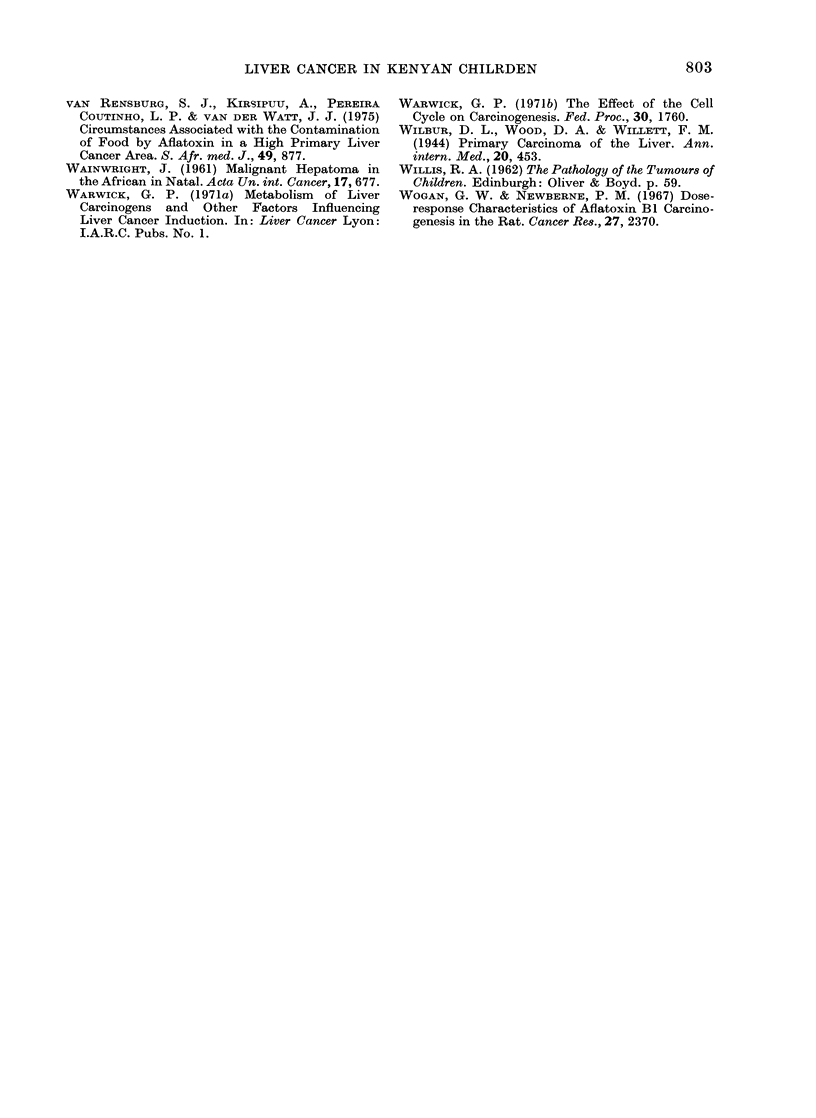# Primary cancer of the liver in Kenyan children.

**DOI:** 10.1038/bjc.1977.264

**Published:** 1977-12

**Authors:** H. M. Cameron, G. P. Warwick

## Abstract

**Images:**


*Br. J. Cancer* (1977) **36**, 793

### PRIMARY CANCER OF THE LIVER IN KENYAN CHILDREN

H. M. CAMERON* AND G. P. WARWICK†

*From the Department of Pathology, University of Nairobi, Kenya*

Received 31 December 1976 Accepted 15 July 1977

**Summary.—In 9 years in Kenya, 34 examples of primary liver cancer were diagnosed in patients in the first two decades of life. This represents 4·7% of all liver cancers during this period. The larger proportion (29) were hepatocellular carcinoma. In the second decade, there was a notable association with macronodular cirrhosis. Analogy with experimental work suggests that cells in mitotic cycle may be more vulnerable to the effect of environmental carcinogens.**

**Five examples of hepatoblastoma were identified at ages from 2 months to 14 years; none showed the features of "mixed" tumours. The ratio of hepatoblastoma to hepatocellular carcinoma was the reverse of that found in other large series of juvenile hepatic tumours.**

**The histopathological features of these tumours are described and problems of their classification are discussed.**

IT has been said that there is a "strange absence" of primary cancer of the liver in children in parts of the world in which it is common in adults (Edmondson, 1956; Fraumeni, Miller and Hill, 1968). Since these areas, which include Africa south of the Sahara, are mostly in countries with recently developed and inadequate medical resources, one should be cautious about accepting comparisons with more generously endowed communities. However, the statement is not altogether supported by the small number of papers from sub-Saharan Africa in which relevant data are given (Table I). Davies (1955) thought that the apparent dearth of juvenile cases was probably the result of "lack of observation or reporting", and this appears to be confirmed by Anthony's later finding in Uganda (1973) of 11 examples of liver cell carcinoma in the under-twenties. As one might expect, even larger numbers have been reported from southern Africa, notably Lourenço Marques (Prates, 1961). In Kenya, although liver cancer apparently does not reach the very high incidence found in Mozambique (Torres, Purchase and van der Watt, 1970), it comes fifth in the table of relative frequency ratios (Linsell, 1967). Investigation of liver disease in early life may reasonably be expected to yield some clues to aetiological factors and, with this in mind, we have reviewed liver disease in Kenya in patients in the first two decades of life (Bowry and Cameron, 1976). This revealed a considerable number of cases of primary cancer of the liver.

### MATERIALS AND METHODS

Kenya has a population of almost 11 million (1969 census). The histopathological laboratory of the University of Nairobi provides a diagnostic service for almost the the entire country (a small proportion of specimens is dealt with at the Nairobi hospital, the Aga Khan Hospital, Nairobi and the Provincial Hospital at Kisumu). Provision of medical services (and particularly, availability of sophisticated investi-

Present addresses: * Department of Pathology, University of Edinburgh.
† International Union against Cancer, Box 400, 1211 Geneva.

TABLE I.—*Juvenile Cases of Primary Liver Cancer Reported from African Centres*

| Author | Country | Total | Cases of primary liver cancer in children | Age range (yrs) |
|---|---|---|---|---|
| Davies (1955) | Uganda | 63 | 0 | |
| Steiner and Davies (1957) | Uganda | 90 | 3 | 0–19 |
| Wainwright (1961) | S. Africa (Natal) | 120 | 17 | 0–19 |
| Prates (1961) | Mozambique (Lourenço Marques) | 526 | 107 | 0–20 (7 < 10) |
| Gelfand, Castle and Buchanan (1972) | Rhodesia | 90 | 3 | 0–20 |
| Anthony (1973) | Uganda | 263 | 11 | 0–20 |
| Cameron and Warwick (this paper) | Kenya | 716 | 34 | 0–20 |

gations such as biopsy) is limited and uneven. Necropsies are performed regularly at the teaching centre, the Kenyatta National Hospital; elsewhere, they are carried out infrequently, and the tissues are submitted to the laboratory in Nairobi for diagnosis. We reviewed all examples of liver disease at ages 0–20 years diagnosed histologically on biopsy or necropsy material received at this laboratory over the years 1965–1973 inclusive. All tumours of the liver were re-examined microscopically.

Two main histological forms of primary liver cancer are found in children:

1. A tumour which is indistinguishable from adult hepatocellular carcinoma;
2. An embryonic tumour analogous to the nephroblastoma. Willis (1962) uses *hepatoblastoma* for all such embryonic tumours, and he sub-divides them into "embryonic hepatomas" which contain only embryonic liver tissue, "mixed tumours", which include bone or cartilage, and the rare "rhabdomyoblastic mixed tumours".

We did not see any examples with mesenchymal elements, and for the purposes of this paper we confined ourselves to the two main categories: hepatocellular carcinoma and hepatoblastoma. Even on this simple classification, allocation of occasional tumours is uncertain and arbitrary, and the sole criterion may be whether the tumour cells are recognizably of parenchymal type or not (Shorter *et al.*, 1960).

### RESULTS

Thirty-four examples of primary liver cancer were identified. During this 9-year period, the total number of cancers in all sites and at all ages diagnosed histologically was 12,674; of these 716 (5·6%) were primary hepatic neoplasms. The 34 juvenile examples made up 4·7% of all liver cancers. Of these 29 were hepatocellular carcinoma and 5 hepatoblastoma.

### *Hepatocellular carcinoma*

Details of the 29 cases are shown in Table II. Two occurred below the age of 10 years and the younger one (Case 1) was only 2 months old (Fig. 1). In this, the tumour cells, although mostly small, bore a close resemblance to hepatic parenchymal cells and in some parts were arranged in trabeculae. The remaining cases in the two decades showed the range of features commonly seen in tumours of adults (Figs. 2 and 3). Attempts at formation of ducts and acini were seen in some; in Case 19 (Fig. 4) parts resembled cholangiocarcinoma; it was clear from its major component, however, that it was essentially hepatocellular. Haemopoiesis was present in the tumour in Cases 1 and 4, and in both tumour and the neighbouring parenchyma in Case 13 (Fig. 5). In two cases (11 and 12) there was associated hepatic schistosomiasis.

In 12 specimens there was sufficient non-neoplastic liver for histological assessment, and 8 of these showed macronodular cirrhosis. All 8 patients were between the ages of 12 and 20 years.

TABLE II.—*Hepatocellular Carcinoma in Age Group 0–20 Years*

| Case | Age (yrs) | Sex | Tribe | Cirrhosis* | Nature of specimen |
|---|---|---|---|---|---|
| 1 | 2/12 | F | — | — | Necropsy |
| 2 | 9 | F | Meru | I | Open biopsy |
| 3 | 12 | M | Kikuyu | I | Needle biopsy |
| 4 | 12 | F | Tugen | — | Necropsy |
| 5 | 12 | M | Kamba | + | Needle & necropsy |
| 6 | 12 | M | Luhya | I | Open |
| 7 | 12 | M | Kikuyu | I | Open |
| 8 | 13 | M | Luo | I | Open |
| 9 | 13 | M | Kamba | I | Needle |
| 10 | 13 | M | Kikuyu | — | Needle & necropsy |
| 11 | 14 | F | Luo | + | Open |
| 12 | 15 | F | Kikuyu | — | Necropsy |
| 13 | 15 | M | Kikuyu | + | Open |
| 14 | 15 | M | Kikuyu | I | Needle |
| 15 | 15 | M | Luhya | I | Open |
| 16 | 16 | M | Marakwet | I | Open |
| 17 | 16 | F | Luo | I | Needle |
| 18 | 16 | F | Kikuyu | + | Open |
| 19 | 16 | F | Elgeyo | + | Necropsy |
| 20 | 18 | M | Kikuyu | I | Needle |
| 21 | 18 | M | Kamba | I | Needle |
| 22 | 18 | M | Luo | I | Needle |
| 23 | 19 | M | Kikuyu | I | Needle |
| 24 | 19 | M | Kamba | + | Necropsy |
| 25 | 20 | M | Duruma | I | Open |
| 26 | 20 | F | Kikuyu | I | Needle |
| 27 | 20 | M | Meru | + | Necropsy |
| 28 | 20 | M | Somali | I | Necropsy |
| 29 | 20 | M | Kikuyu | + | Necropsy |

*+ = Cirrhosis.
— = No evidence of cirrhosis.
I = Insufficient for assessment.

FIG. 1.—Case 1: Hepatocellular carcinoma in a girl of 2 months. Trabecular arrangement of tumour cells of parenchymal type. Cirrhosis absent. H. and E. × 275.

*Hepatoblastoma*

In all 5 cases (Table III) the tumour was made up of sheets of uniform small cells with scanty lightly basophilic cytoplasm and a relatively large nucleus (Fig. 6). The chromatin of the latter was "open" and granular, and there was a prominent nuclear membrane. Mitotic activity varied, being very high in Case 30 and low in Case 31. In only one case (Case 32) was there an attempt at duct formation. Haemopoiesis was seen within the tumour in Case 32, but was not a prominent feature. The diagnosis in Case 30 was difficult because the biopsy specimen was very small, but the presence of bile in tumour cells confirmed that they were of hepatic type.

Case 34 was the most difficult to substantiate and is therefore presented in greater detail. This was a 14-year-old Somali boy who had been troubled by epigastric pain "for years". He experienced dysphagia and had occasional haematemeses. On admission to Meru Hospital, he was extremely anaemic and emaciated but was not jaundiced. He was in a state of shock, and vomited dark brown fluid.

FIG. 2.—Case 7: Hepatocellular carcinoma in a boy of 12 years. H. and E. × 450.

FIG. 3.—Case 18: Hepatocellular carcinoma—clear-cell type in a girl of 16 years. The liver was cirrhotic. H. and E. × 450.

Attempts to counteract this with blood transfusion were unsuccessful, and he died 12 h after admission.

At necropsy, a pale liver was found containing 5 or 6 round tumour masses 4–5 cm in diameter. Both lobes were affected. Otherwise the liver was normal and there was no cirrhosis. The spleen was enlarged but in other respects normal. No other tumour was found in a full post-

FIG. 4.—Case 19: Hepatocellular carcinoma in a girl of 16 years. Parts of the tumour have a ductular structure and resemble cholangiocarcinoma. The liver was cirrhotic. H. and E. × 450.

FIG. 5.—Case 13: Hepatocellular carcinoma in a boy of 13 years. There is extramedullary haemopoiesis. The liver was cirrhotic. H. and E. × 450.

TABLE III.—*Hepatoblastoma in Age Groups 0–20 Years*

| Case | Age (yrs) | Sex | Tribe | Nature of specimen |
|---|---|---|---|---|
| 30 | 2/12 | M | Kalenjin | Needle |
| 31 | 9/12 | F | Meru | Open |
| 32 | 2 | M | Kipsigis | Open |
| 33 | 5 | M | Kamba | Open |
| 34 | 17 | M | Somali | Necropsy |

mortem examination. On histological examination, some shrinkage artefact was seen as a result of postmortem autolysis, but preservation of cells was good. The tumour was composed of uniform small cells with deeply staining, granular nuclei (Fig. 7). They were arranged in large "packets" but had no trabecular or ductular pattern. There was no cirrhosis of the non-neoplastic liver.

### DISCUSSION

### *Histology*

Most hepatocellular carcinomas, whether in adults or children, are sufficiently well differentiated to be easily recognized. Similarly, the typical embryonic hepatoblastoma poses little diagnostic problem, particularly when it is of "mixed" type. When there is no "foreign" mesenchymal element, distinction from hepatocellular carcinoma may be arbitrary and subjective. Edmondson (1956) found every grade of transition between the two, and Willis (1962) acknowledged that some tumours in older children are difficult to classify. The distinction between the two tumours may not be entirely academic, since it is likely that the factors which produce a truly embryonic tumour differ from those which give rise to a carcinoma of adult type. Some workers have attempted to make more elaborate classifications (Ishak and Glunz, 1967; Kasai and Watanabe, 1970) but our experience, in keeping with the findings of Edmondson (1956) Willis (1962) and Sinniah, Campbell and Colebatch (1974), is that there is a "grey zone" between the occasional poorly differentiated hepatocellular carcinoma and the purely epithelial hepatoblastoma. It is unsatisfactory to have no more objective criterion than the degree to which tumour cells resemble hepatic parenchyma, but we find that for practical diagnostic purposes there is often no other.

FIG. 6.—Case 31: Hepatoblastoma in a 9-month-old girl. H. and E. × 450.

FIG. 7.—Case 34: Hepatoblastoma in a boy of 14 years. H. and E. × 450.

It has been said that extra-medullary haemopoiesis is a feature of hepatoblastoma (Misugi *et al.*, 1967) and Shiødt (1970) takes this as favouring the embryonic nature of the tumour. We found it in 1 of our 5 cases but also in 3 hepatocellular carcinomas, and therefore of no diagnostic help. This is hardly surprising, particularly in young anaemic children.

### *Age*

The ages of the 34 cases of primary cancer of the liver ranged from 2 months to 20 years. Comparison of age distribution with other series is difficult because: (a) the numbers in any series are small; (b) available statistics (Doll, Muir and Waterhouse, 1970) do not distinguish between hepatocellular carcinoma and hepatoblastoma; (c) in countries like Kenya, with a scattered population of about 11 million, the provision of medical services is uneven, and it is likely that many cases of neoplasia go undiagnosed; and (d) the available data do not permit any realistic assessment of population frequencies.

*Hepatoblastoma.*—Most hepatoblastomas are identified in the first two years of life (Fraumeni *et al.*, 1968; Misugi *et al.*, 1967; Ishak and Glunz, 1967; Keeling, 1971) and in the series of Misugi *et al.* (1967) there was a clear separation of their 19 examples of embryonal tumours from the 5 "hepatomas". The former were all less than 3 years of age and the latter were more than 6, no tumours being found in the intervening years. Similarly Ishak and Glunz (1967) described 35 hepatoblastomas, all in children aged less than 40 months; of their 12 hepatocarcinomas, only one was less than 5 years. Nevertheless, distinction by age is not always clear-cut: occasional examples of hepatocellular carcinoma occur in the younger age group, and some cases of hepatoblastoma are found after the age of 3. Milman and Grayzel (1951) describe a "mixed" tumour in a boy of 6, and Willis' (1962) oldest case was 7 years old. In a series of 16 cases (Sinniah *et al.*, 1974) 3 were aged between 6 and 10 years. We agree with Ishak and Glunz (1967) in doubting whether some examples of

"mixed" tumours reported in adults are related to hepatoblastoma, but since this is an embryonic tumour, one might expect to find, as with nephroblastoma, occasional examples in adults (Barnett, Erickson and Halpert, 1958); some, indeed have been described in elderly patients (Alexander, 1961; Carter, 1969).

Of the present group of 5 hepatoblastomas, 3 occurred within the first 2 years of life, and one was 5 years. The last was 14 years old, and required careful scrutiny, both because of the age and because it was multinodular. The latter feature does not exclude the diagnosis (Bigelow and Wright, 1953; Shorter *et al.*, 1960; Ishak and Glunz, 1967) and the histological resemblance to the other 4 cases persuaded us it was a hepatoblastoma.

*Hepatocellular carcinoma.*—We found two examples of hepatocellular carcinoma in the first decade, one at 2 months. The latter patient had a distended abdomen and a large hard liver since birth, and this was thought to be a congenital neoplasm similar to that described by Wilbur, Wood and Willett (1944). McDougal and Gatzimos (1957) present 5 cases of hepatocellular carcinoma in patients below the age of 5 years, and 2 of these were less than one year old; in the series of 11 cases by Shorter *et al.* (1960), 4 were less than 10 years old and 2 of these were in the first year of life.

### *Sex*

The series of 29 hepatocellular carcinomas shows the marked male predominance (20:9) which is well recognized in adult cases. The numbers of hepatoblastoma are too small to show whether this is a regular feature.

### *Geographical variation*

This survey has been carried out in a country with limited medical facilities which militate against early investigation and all too often preclude follow-up. Any comparison with more sophisticated centres is therefore hazardous, particularly when dealing with lesions as uncommon as childhood neoplasms of the liver. One can however make some valid observations.

1. Our 34 cases were collected over a period of 9 years. This can be compared with figures from some major centres: 11 cases in 53 years at the Mayo Clinic (Shorter *et al.*, 1960); 24 cases in 18 years at the Children's Hospital, Columbus, Ohio (Misugi *et al.*, 1967); 47 from the Registries of Pediatric and Hepatic Pathology at the Armed Forces Institute of Pathology over a period of more than 40 years (Ishak and Glunz, 1967); 46 since 1926 at Great Ormond Street, London (Keeling, 1971); 30 in 22 years at the Hospital for Sick Children, Toronto (Ein and Stephens, 1974) and 20 in 22 years at the Royal Children's Hospital, Melbourne (Sinniah *et al.*, 1974).

2. In five large series (Ishak and Glunz, 1967; Misugi *et al.*, 1967; Kasai and Watanabe, 1970; Keeling, 1971; and Ein and Stephens, 1974), hepatoblastomas outnumber hepatocellular carcinomas by 169 to 38. The proportions are reversed in the Kenyan series, there being 29 hepatocellular carcinomas and 5 hepatoblastomas. One might postulate that the incidence of hepatoblastoma, like that of nephroblastoma, is unlikely to show much geographical variation. The proportionate increase in hepatocellular carcinoma would then point to the existence of some local carcinogenic factors. The fact that 11 cases occurred in patients below the age of 15 years indicates that such influences start early in life.

3. In neighbouring Uganda, the proportion of liver cancers found at age 20 or less (4·2% Anthony, personal communication) is similar to the 4·7% found in Kenya, and this confirms that the "strange absence" of juvenile cases (Edmondson, 1956) is an illusion as far as these high-frequency areas are concerned.

### *Pathogenesis of hepatocellular carcinoma*

It seems that cirrhosis is seldom associated with hepatocellular carcinoma in very young children in the first decade of life, although individual examples have been reported (Jones, 1960). However, in this series, the association is a notable feature after the age of 10; macronodular cirrhosis was found in 8/12 in which there was adequate tissue for assessment.

The precise role of cirrhosis in hepatic carcinogenesis is uncertain, but the constant high association with hepatocellular carcinoma seen in all parts of the world suggests an important clue to the pathogenesis of the tumour. It is not known, however, whether the association is coincidental or causative, whether the cirrhotic liver is intrinsically prone to neoplasia or simply more vulnerable to the action of environmental carcinogens. Epidemiological and experimental work suggests an important role for such carcinogens. In areas where liver cancer is common, both viruses and chemical substances have been proposed as possible oncogenic agents.

In Africa south of the Sahara, the hepatitis B surface antigen (HBsAg) is found widely in the African population (Parker, Muiruri and Preston, 1971; Bagshawe and Nganda, 1973; Lowenthal *et al.*, 1973). It is not only associated with viral hepatitis, but is found in a large proportion of cases with chronic hepatitis and cirrhosis (Anthony *et al.*, 1972; Kew *et al.*, 1974). The proportion of positives in adult hepatocellular carcinoma varies widely in different series: from 1·3% to 65% (Bagshawe, 1975). It has been suggested that the hepatitis B virus may "set the scene" for liver cancer by producing cirrhosis or, alternatively, that it may itself on occasions be oncogenic.

Much attention has been paid recently to the possibility that chemical carcinogens in the environment may be responsible for the frequency of liver cancer in Africa. Aflatoxin B1 has repeatedly been identified in food, and surveys show not only that man does ingest this carcinogen, but that there is a correlation between the intake of aflatoxin and the incidence of liver-cell cancer (Peers and Linsell, 1973; van Rensburg *et al.*, 1975). Aflatoxin has been identified in human milk (Burton, 1971) and experimental studies show that it can be transferred to offspring in maternal milk, and result in tumours in later life (Mohr and Althoff, 1971; Grice, Moodie and Smith, 1973).

The very high degree of association of hepatocellular carcinoma with cirrhosis should not be taken as evidence that the latter is itself premalignant. A sizeable proportion of tumours develop in the absence of cirrhosis, and this appears to include those occurring in very young children. Experimental evidence shows no parallel relationship between the cirrhogenic and the carcinogenic effects of chemicals. Some agents such as carbon tetrachloride are strongly cirrhogenic but are weak carcinogens (Warwick, 1971*a*). With others (including aflatoxin B1) the reverse is true (Wogan and Newberne, 1967). Thus, although evidence from man suggests a central role for cirrhosis in hepatic carcinogenesis, much experimental work contradicts this.

A more important factor may be the intensity of mitotic activity in the liver at the time of administration of a carcinogen. Newborn and young animals are often highly sensitive to hepatocarcinogens (Della Porta and Terracini, 1969). The same is true of adult animals whose livers are regenerating following partial hepatectomy (Craddock and Frei, 1974; Warwick, 1971*b*) or chemical damage (Craddock, 1976). It would seem that cells in cycle are more vulnerable to the action of hepatocarcinogens. If so, this could be relevant to human liver cell cancer in two circumstances: (a) when exposure occurs in early life, during active liver growth (if environmental carcinogens were responsible for the tumours seen in this study they must have been encountered very early); (b) in adults whose livers show an abnormally high level of regenerative activity resulting from diverse liver diseases. On this basis one might postulate that the association between cirrhosis and liver cancer is accounted for by the regenerative activity which is a notable feature of cirrhosis, but which it shares with other diseases in which hepatic cells die.

We wish to thank Dr Say van der Werf of Meru Hospital, who kindly supplied us with details of the autopsy on Case 34.

### REFERENCES

ALEXANDER, M. K. (1961) A Mixed Tumour of the Liver in an Adult. *J. Path. Bact.*, **82**, 217.

ANTHONY, P. P. (1973) Primary Carcinoma of the Liver: a Study of 282 Cases in Ugandan Africans. *J. Path.*, **110**, 37.

ANTHONY, P. P., VOGEL, C. L., SADIKALI, F., BARKER, L. F. & PETERSON, M. R. (1972) Hepatitis-associated Antigen and Antibody in Uganda. *Brit. med. J.*, i, 403.

BAGSHAWE, A. F. (1975) Hepatocellular Carcinoma. In: *Modern Trends in Gastroenterology*, Ed. A. E. Read. London: Butterworths. p. 115

BAGSHAWE, A. F. & NGANDA, T. N. (1973) Hepatitis-B Antigen in a Rural Community in Kenya. *Trans. R. Soc. trop. Med. Hyg.*, **67**, 663.

BARNETT, W. H., ERICKSON, E. E. & HALPERT, B. (1958) Embryonic Tumor of the Liver in an Adult. *Cancer, N.Y.*, **11**, 306.

BIGELOW, N. H. & WRIGHT, A. W. (1953) Primary Carcinoma of the Liver in Infancy and Childhood. *Cancer, N.Y.*, **6**, 170.

BOWRY, T. & CAMERON, H. M. (1976) Liver Disease in Early Life in Kenya. *Proc. R. Soc. trop. Med. Hyg.*, **70**, 439.

BURTON, B. T. (1971) Nutrition Research Notes from Niamid. *Nutrition Notes*, p. 5.

CARTER, R. (1969) Hepatoblastoma in the Adult. *Cancer, N.Y.*, **23**, 191.

CRADDOCK, V. M. (1976) In: *Liver Cell Cancer*, Ed. H. M. Cameron, C. A. Linsell and G. P. Warwick. Amsterdam: Biochemical Press, V. B. p. 153.

CRADDOCK, V. M. & FREI, J. V. (1974) Induction of Liver Cell Adenomata in the Rat by a Single Treatment with N-methyl-N-nitrosourea given at Various Times after Partial Hepatectomy. *Br. J. Cancer*, **30**, 503.

DAVIES, J. N. P. (1955) Human Implications. Primary Carcinoma of the Liver in Africans. *J. natn. Cancer Inst.*, **15** (Suppl.), 1637.

DELLA PORTA, G. & TERRACINI, B. (1969) Chemical Carcinogenesis in Infant Animals. *Tumor Res.*, **11**, 334.

DOLL, R., MUIR, C. & WATERHOUSE, J. (1970) *Cancer Incidence in Five Continents.* Vol. 2. U.I.C.C. Berlin: Springer-Verlag. p. 85.

EDMONDSON, H. A. (1956) Differential Diagnosis of Tumors and Tumor-like Lesions of Liver in Infancy and Childhood. *J. Dis. Child.*, **91**, 168.

EIN, S. H. & STEPHENS, C. A. (1974) Malignant Liver Tumours in Children. *J. Pediat. Surg.*, **9**, 491.

FRAUMENI, J. F., MILLER, R. W. & HILL, J. A. (1968) Primary Carcinoma of the Liver in Childhood. *J. natn. Cancer Inst.*, **40**, 1087.

GELFAND, M., CASTLE, W. M. & BUCHANAN, W. M. (1972) Primary Carcinoma of the Liver (Hepatoma) in Rhodesia. *S. Afr. med. J.*, **46**, 527.

GRICE, H. C., MOODIE, C. A. & SMITH, D. C. (1973) The Carcinogenic Potential of Aflatoxin or its Metabolites in Rats from Dams Fed Aflatoxin Pre- and Post-partum. *Cancer Res.*, **33**, 262.

ISHAK, K. G. & GLUNZ, P. R. (1967) Hepatoblastoma and Hepatocarcinoma in Infancy and Childhood. *Cancer, N.Y.*, **20**, 396.

JONES, E. (1960) Primary Carcinoma of the Liver with Associated Cirrhosis in Infants and Children. *Archs Path.*, **70**, 19/5.

KASAI, M. & WATANABE, I. (1970) Histological Classification of Liver Cell Carcinoma in Infancy and Childhood and its Clinical Evaluation. A Study of 70 Cases Collected in Japan. *Cancer, N.Y.*, **25**, 551.

KEELING, J. W. (1971) Liver Tumours in Infancy and Childhood. *J. Path.*, **103**, 69.

KEW, M. C., GEDDES, E. W., MACNAB, G. M. & BERSOHN, I. (1974) Hepatitis-B Antigen and Cirrhosis in Bantu Patients with Primary Liver Cancer. *Cancer, N.Y.*, **34**, 539.

LINSELL, C. A. (1967) Cancer in Kenya. In: *Cancer in Africa*. Ed. P. Clifford, C. A. Linsell and G. L. Timms. Nairobi: E. Afr. Pub. Hse. p7.

LOWENTHAL, M., BANATVALA, J. E., CHRYSTIE, I. L., JONES, I. G., NAG, J., MOHELSKY, V. & HUTT, M. S. R. (1973) Australia Antigen in Liver Disease in Zambia. *Trop. geogr. Med.*, **25**, 39.

MCDOUGAL, R. A. & GATZIMOS, C. D. (1957) Primary Carcinoma of the Liver in Infants and Children. *Cancer, N.Y.*, **10**, 678.

MILMAN, D. H. & GRAYZEL, D. M. (1951) Mixed Tumor of the Liver. *Am. J. Dis. Child.*, **81**, 408.

MISUGI, K., OKAJIMA, H., MISUGI, N. & NEWTON, W. A. (1967) Classification of Primary Malignant Tumors of the Liver in Infancy and Childhood. *Cancer, N.Y.*, **20**, 1760.

MOHR, U. & ALTHOFF, J. (1971) Carcinogenic Activity of Aliphatic Nitrosamines *via* the Mother's Milk in the Offspring of Syrian Golden Hamsters. *Proc. exp. Biol. Med.*, **136**, 1007.

PARKER, A. M., MUIRURI, K. L. & PRESTON, J. K. (1971) Hepatitis-associated Antigen in Blood Donors in Kenya. *E. Afr. med. J.*, **48**, 470.

PEERS, F. G. & LINSELL, C. A. (1973) Dietary Aflatoxins and Liver Cancer: a Population Based Study in Kenya. *Br. J. Cancer*, **27**, 473.

PRATES, M. D. (1961) Cancer and Cirrhosis of the Liver in the Portuguese East African with Special Reference to the Specific Age and Sex Rates in Lourenço Marques. *Acta Un. int. Cancr.*, **17**, 718.

SCHIØDT, T. (1970) Hepatoblastoma and Hepatocarcinoma in Infancy and Childhood. *Acta path. microbiol. scand.*, **212**, 181.

SHORTER, R. G., BAGGENSTOSS, A. H., LOGAN, G. B. & HALLENBECK, G. A. (1960) Primary Carcinoma of the Liver in Infancy and Childhood. *Pediatrics*, **25**, 191.

SINNIAH, D., CAMPBELL, P. E. & COLEBATCH, J. H. (1974) Primary Hepatic Cancer in Infancy and Childhood. In: *Progress in Pediatric Surgery*. Vol. 7. Ed. P. P. Rickam, W. Ch. Hecker and J. Prévot. Munich: Urban and Schwarzenberg. p. 141.

STEINER, P. E. & DAVIES, J. N. P. (1957) Cirrhosis and Primary Liver Carcinoma in Uganda Africans. *Br. J. Cancer*, **11**, 523.

TORRES, I. O., PURCHASE, I. F. H. & VAN DER WATT, J. J. (1970) The Aetiology of Primary Liver Cancer in the Bantu. *J. Path.*, **102**, 163.

van Rensburg, S. J., Kirsipuu, A., Pereira Coutinho, L. P. & van der Watt, J. J. (1975) Circumstances Associated with the Contamination of Food by Aflatoxin in a High Primary Liver Cancer Area. *S. Afr. med. J.*, **49**, 877.

Wainwright, J. (1961) Malignant Hepatoma in the African in Natal. *Acta Un. int. Cancer,* **17**, 677.

Warwick, G. P. (1971*a*) Metabolism of Liver Carcinogens and Other Factors Influencing Liver Cancer Induction. In: *Liver Cancer* Lyon: I.A.R.C. Pubs. No. 1.

Warwick, G. P. (1971*b*) The Effect of the Cell Cycle on Carcinogenesis. *Fed. Proc.*, **30**, 1760.

Wilbur, D. L., Wood, D. A. & Willett, F. M. (1944) Primary Carcinoma of the Liver. *Ann. intern. Med.*, **20**, 453.

Willis, R. A. (1962) *The Pathology of the Tumours of Children.* Edinburgh: Oliver & Boyd. p. 59.

Wogan, G. W. & Newberne, P. M. (1967) Dose-response Characteristics of Aflatoxin B1 Carcinogenesis in the Rat. *Cancer Res.*, **27**, 2370.